# Personality Traits and Body Mass Index in a Korean Population

**DOI:** 10.1371/journal.pone.0090516

**Published:** 2014-03-05

**Authors:** Unjin Shim, Han-Na Kim, Seung-Ju Roh, Nam H. Cho, Chol Shin, Seungho Ryu, Yeon-Ah Sung, Hyung-Lae Kim

**Affiliations:** 1 Department of Internal Medicine, Seoul Seonam Hospital, Ewha Womans University Medical Center, Seoul, Korea; 2 Department of Biochemistry, Ewha Womans University, School of Medicine, Seoul, Korea; 3 Department of Preventive Medicine, Ajou University, School of Medicine, Suwon, Korea; 4 Division of Pulmonary and Critical Care Medicine, Department of Internal Medicine, Korea University Ansan Hospital, Ansan, Korea; 5 Department of Occupational Medicine, Kangbuk Samsung Hospital, Sungkyunkwan University, School of Medicine, Seoul, Korea; 6 Department of Internal Medicine, Ewha Womans University, School of Medicine, Seoul, Korea; Newcastle University, United Kingdom

## Abstract

**Background:**

Overweight and obesity is a serious problem worldwide related to cardiovascular and other diseases. Personality traits are associated with the abnormal body mass indices (BMIs) indicative of overweight and obesity. However, the links between personality traits and BMI have been little studied in Korea.

**Methods:**

We evaluated the association between personality traits and BMI in men and women using the rural Ansung and urban Ansan cohort from the Korean Genome Epidemiology Study, and the Kangbuk Samsung Hospital Cohort Study datasets. A shorter version of the original Revised Neuroticism-Extroversion-Openness Personality Inventory (NEO-PI-R) was used to measure the five-factor model of personality (neuroticism, extraversion, openness to experience, agreeableness, and conscientiousness).

**Results:**

Data from a total of 1,495 men (mean age 60.0±9.8 years; mean BMI 24.3±3.0 kg/m^2^) and 2,547 women (mean age 47.0±15.5 years; mean BMI 22.8±3.4 kg/m^2^) were included in the analysis. Compared with the normal weight groups, overweight and obese men scored higher on openness to experience and lower on conscientiousness. Overweight and obese women scored lower on neuroticism and openness to experience and higher on agreeableness. Extraversion was positively associated with BMI in men (β = 0.032, *P*<0.05). BMI and waist circumference were significantly increased in individuals who were less dutiful. In women, neuroticism was inversely associated with BMI (β = −0.026, *P*<0.05). Openness to experience was negatively, and agreeableness was positively, associated with BMI (openness to experience: β = −0.072, agreeableness β = 0.068) and waist circumference (openness to experience: β = −0.202, agreeableness: β = 0.227) (*P*<0.05).

**Conclusion:**

Personality traits were associated with underweight, overweight, and obesity in men and women. Increased understanding of the underlying factors contributing to this association will aid in the prevention and treatment of abnormal BMI.

## Introduction

Overweight and obesity are serious problems worldwide and are associated with diabetes and cardiovascular disease [1], [2]. They are also associated with psychiatric disorders. For example, there is a U-shaped association between depression and body mass index (BMI) in underweight, normal, overweight, and obese groups [3], [4]. Personality traits are commonly evaluated using the Revised Neuroticism-Extroversion-Openness Personality Inventory (NEO PI-R), which measures the five-factor model (FFM) of personality: neuroticism, extraversion, openness to experience, agreeableness, and conscientiousness [5]. Many studies have used the FFM to identify personality traits linked with overweight and obesity [6]–[10]. Neuroticism, the tendency to experience negative emotions, is related to emotional eating, increased hunger susceptibility and disinhibition. Neurotic individuals are more likely to be impulsive and depressed [7], [11]. Neuroticism is associated with all-cause mortality in the elderly [12]. Conscientiousness (i.e., the tendency to be self-controlled, organized, and follow principles) is a positive trait and protects against the development of obesity [13]. Higher neuroticism and lower conscientiousness are associated with increased BMI values, higher adipose levels, and greater leptin resistance [6], [8]. Impulsiveness, a facet of neuroticism, is higher in individuals with a larger waist circumference, and individuals with lower conscientiousness have elevated triglyceride and decreased HDL cholesterol levels [10], [14]. Higher BMI is associated with low extraversion, low openness to experience, and high agreeableness, but only a limited number of studies have been published on the relationship between BMI and these traits [15]–[17].

Recent studies have emphasized the contribution of lower conscientiousness to the development of obesity and related diseases, including metabolic syndrome and diabetes [9], [13], [18]. However, the majority of the studies of personality traits and BMI have been performed in populations from Western countries. Data on Asian populations are relatively rare. One large population study in Japan used the Eysenck Personality Questionnaire and found that overweight and obese groups score lower in neuroticism and higher in extraversion [15]. These results are inconsistent with previous large population studies that found that neuroticism is higher in overweight/obese groups [6], [7], [10], [19]. Because only a limited number of reports of the relationships between personality traits and abnormal BMI have been published for Asian populations (e.g., Korea), the results of the Japan study cannot be compared with other Asian studies. Therefore, the objective of this study was to evaluate the association between personality traits and abnormal BMI in a Korean population. To our knowledge, this is the first study to use the FFM to examine the association between personality traits and underweight, overweight, and obesity in a Korean population.

## Research Design and Methods

### Study Subjects

We used the datasets from the rural Ansung and the urban Ansan cohorts of the Korean Genome Epidemiology Study (KoGES) [20] and from the cohort study of Kangbuk Samsung hospital (KSCS) to evaluate the association between personality traits and abnormal BMI in men and women. KSCS is a large prospective cohort study that is being used to evaluate the natural histories, prognoses, and genetic and environmental determinants of a wide range of health traits and diseases among adult Korean men and women.

Data from a total of 1,495 men and 2,547 women that participated in these cohort studies were included in the analysis. Hospital personnel recorded the anthropometric measurements of all participants. Weight and height were measured with the subjects wearing light clothing and no shoes. BMI was also calculated (kg/m^2^). Waist circumference was measured to the nearest 0.1 cm on bare skin during mid-respiration at the narrowest indentation between the 10^th^ rib and the iliac crest. Participants were divided into four groups: underweight (BMI <18.5 kg/m^2^), normal weight (BMI 18.5–22.9 kg/m^2^), overweight (BMI 23–24.9 kg/m^2^), and obese (BMI ≥25.0 kg/m^2^). We used cut-off points recommended for Asians by the International Association for the Study of Obesity (IASO), the International Obesity Task Force (IOTF), and the World Health Organization (WHO) to define overweight and obesity [21], [22]. To obtain accurate BMI results, and to prevent the introduction of recall bias, we excluded data from participants who self-reported their weight and height.

Personality traits were assessed using the Korean short version (100 items) of the original NEO PI-R. The questionnaire was designed to measure the FFM of personality: neuroticism, extraversion, openness to experience, agreeableness, and conscientiousness (PSI consulting Corp., Seoul, Korea) [5], [23], [24]. Each factor consisted of a subset of six variables. The neuroticism factor included the variables anxiety, angry hostility, depression, self-consciousness, impulsiveness, and vulnerability. The extraversion factor included the variables warmth, gregariousness, assertiveness, activity, excitement-seeking, and positive emotions. Openness to experience included fantasy, aesthetics, feelings, actions, ideas, and values. The factor agreeableness consisted of trust, straightforwardness, altruism, compliance, modesty, and tender-mindedness. The factor conscientiousness included the variables competence, order, dutifulness, achievement striving, self-discipline, and deliberation.

Socioeconomic status was categorized using the subject’s educational level and income. Educational level included four categories: 1 =  illiterate or up to elementary school, 2 =  middle school, 3 =  high school, 4 =  college or university. Income consisted of eight categories (i.e., 1 was the lowest, and 8 the highest, level of income).

The Institutional Review Board of Ewha Womans University Mokdong Hospital approved this study. Written informed consent was obtained from all participants.

### Statistical Analysis

SAS version 9.1 statistical software (SAS Institute, Cary, NC, USA) was used for data management and statistical analysis. One-way analysis of variance (ANOVA) was used for multiple comparisons of the BMI groups, and post-hoc tests were performed using Fisher’s least significant difference (LSD) method. The variables age, age^2^, and education were used as covariates. Because only limited income data were available for male participants, we did not include income as a covariate. Data were expressed as adjusted means and standard errors.

Multiple linear regression analysis was used to determine the personality traits associated with BMI and waist circumference. Logistic regression analysis was used to analyze personality traits predicting underweight, overweight, and obesity. All *P-*values were two-tailed, and statistical significance was defined as a *P-*value <0.05.

## Results

The mean age and BMI were 60.0±9.8 years (range 21–103) and 24.3±3.0 kg/m^2^ in men and 47.0±15.5 years (range 19–93) and 22.8±3.4 kg/m^2^ in women. Twenty two (1.5%) men and 273 women (10.7%) were underweight, 407 (27.2%) and 1,452 (57.0%) were normal weight, 410 (27.4%) and 363 (14.3%) were overweight, and 656 (43.9%) and 459 (18.0%) were obese (Table 1).

**Table 1:** Clinical characteristic of subjects.

Characteristic	Male(n = 1,495)	Female(n = 2,547)
Age (yrs)	60.0±9.8	47.0±15.5
BMI (kg/m^2^)	24.3±3.0	22.8±3.4
Waist circumference (cm)	83.6±8.9	79.2±10.0
Weight status (n, %)		
Underweight	22 (1.5)	273 (10.7)
Normal weight	407 (27.2)	1,452 (57.0)
Overweight	410 (27.4)	363 (14.3)
Obese	656 (43.9)	459 (18.0)

Data are presented as mean ± standard deviation.

BMI, body mass index.

The mean ages of the overweight and obese groups were younger in men and were older in women (Tables 2 and 3). Compared with the normal weight groups, the overweight and obese men scored higher on openness to experience and lower on conscientiousness. The overweight and obese women scored lower on neuroticism and openness to experience and higher on agreeableness.

**Table 2:** Adjusted means for personality traits, grouped by body mass index (BMI) categories, men.

Characteristic	BMI (kg/m^2^)			
	<18.5(n = 22)	18.5–22.9(n = 407)	23.0–24.9(n = 410)	≥25(n = 656)
Age (yrs)	67.8 (2.04)	62.9 (0.42)	61.6 (0.40)	59.8 (0.27)
Waist (cm)	67.3 (1.02)*	77.7 (0.28)	84.2 (0.23)*	90.6 (0.25)*
Neuroticism	47.9 (1.45)	49.0 (0.36)	48.9 (0.38)	48.5 (0.28)
Extraversion	59.0 (1.46)	58.7(0.31)	59.2 (0.33)	59.7 (0.28)
Openness	57.0 (0.83)*	56.7 (0.29)	57.1 (0.32)*	57.2 (0.27)*
Agreeableness	64.7 (1.30)	63.6 (0.26)	63.4 (0.27)	63.9 (0.23)
Conscientiousness	61.7 (1.30)*	63.7 (0.31)	63.4 (0.31)*	63.6 (0.27)*
Anxiety (N1)	9.2 (0.51)	9.2 (0.10)	9.0 (0.09)	9.0 (0.07)
Angry hostility (N2)	7.4 (0.35)	7.9 (0.10)	7.9 (0.10)	7.8 (0.78)
Depression (N3)	7.5 (0.37)	7.7 (0.11)	7.6 (0.10)*	7.4 (0.08)*
Self-consciousness (N4)	7.6 (0.50)*	8.0 (0.10)	7.9 (0.09)*	7.9 (0.07)*
Impulsiveness (N5)	8.8 (0.41)	8.3 (0.09)	8.4 (0.09)	8.4 (0.07)*
Vulnerability (N6)	8.0 (0.41)	8.1 (0.09)	8.0 (0.09)	7.8 (0.07)*
Warmth (E1)	10.2 (0.35)	10.2 (0.09)	10.4 (0.08)*	10.6 (0.07)*
Gregariousness (E2)	9.3 (0.63)*	9.5 (0.10)	9.8 (0.09)*	9.7 (0.07)*
Assertiveness (E3)	9.1 (0.53)	9.2 (0.10)	9.5 (0.11)*	9.9 (0.08)*
Activity (E4)	9.1 (0.50)	9.3 (0.09)	9.7 (0.09)	9.7 (0.07)
Excitement-seeking (E5)	10.0 (0.54)	10.2 (0.10)	10.4 (0.10)	10.3 (0.08)
Positive emotions (E6)	9.0 (0.44)	9.2 (0.09)	9.6 (0.09)	9.6 (0.07)
Fantasy (O1)	9.4 (0.41)*	9.3 (0.09)	9.7 (0.08)*	9.8 (0.07)*
Aesthetics (O2)	9.4 (0.54)*	9.7 (0.12)	9.9 (0.12)*	9.9 (0.09)*
Feelings (O3)	10.0 (0.38)*	9.6 (0.08)	9.7 (0.08)*	9.9 (0.06)*
Actions (O4)	9.4 (0.36)	8.5 (0.09)	8.7 (0.09)	8.6 (0.07)
Ideas (O5)	8.7 (0.51)*	8.6 (0.10)	8.9 (0.10)*	8.8 (0.07)*
Values (O6)	11.1 (0.45)	10.4 (0.07)	10.2 (0.08)	10.3 (0.06)*
Trust (A1)	10.6 (0.36)	10.5 (0.08)	10.3 (0.08)	10.6 (0.06)
Straightforwardness (A2)	12.2 (0.50)	11.6 (0.11)	11.1 (0.11)	11.2 (0.79)
Altruism (A3)	11.8 (0.36)	11.0 (0.08)	11.0 (0.08)	11.1 (0.06)
Compliance (A4)	9.7 (0.34)	9.6 (0.08)	9.7 (0.08)	9.4 (0.06)
Modesty (A5)	11.5 (4.30)	11.0 (0.77)	10.8 (0.73)	10.7 (0.06)
Tender-mindedness (A6)	11.5 (0.40)	10.7 (0.08)	10.7 (0.09)	10.7 (0.06)
Competence (C1)	9.4 (0.46)	9.3 (0.08)	9.5 (0.08)*	9.7 (0.07)*
Order (C2)	10.7 (0.42)*	10.5 (0.09)	10.4 (0.09)	10.4 (0.07)*
Dutifulness (C3)	12.2 (0.43)	12.0 (0.08)	12.0 (0.08)*	11.9 (0.06)
Achievement striving (C4)	10.9 (0.43)	10.3 (0.09)	10.4 (0.09)*	10.6 (0.07)*
Self-discipline (C5)	11.0 (0.53)	11.1 (0.08)	11.1 (0.09)	11.1 (0.06)
Deliberation (C6)	10.0 (0.45)*	10.1 (0.09)	10.3 (0.09)	10.1 (0.07)

Data are presented as adjusted mean (standard errors).

All means are controlled for age, age squared and education.

Wilk’s Lambda = 0.979, *P*<0.001, partial η2 = 0.021.

*P<0.05 compared to normal BMI groups (18.5–22.9kg/m^2^).

**Table 3:** Adjusted means for personality traits, grouped by body mass index (BMI) categories, women.

Characteristic	BMI (kg/m^2^)			
	<18.5(n = 273)	18.5–22.9(n = 1452)	23.0–24.9(n = 363)	≥25(n = 459)
Age (yrs)	32.7 (0.36)	36.4 (0.27)	45.8 (0.78)	51.8 (0.68)
Waist (cm)	65.6 (0.28)*	72.9 (0.16)	80.6 (0.33)*	88.3 (0.40)*
Neuroticism	52.9 (0.55)*	51.9 (0.22)	50.7 (0.43)*	50.8 (0.37)*
Extraversion	59.1 (0.45)	60.0 (0.22)	60.5 (0.42)	59.5 (0.38)
Openness	60.2 (0.43)*	60.6 (0.20)	59.5 (0.39)*	58.2 (0.34)*
Agreeableness	61.4 (0.43)*	62.4 (0.17)	63.2 (0.34)*	64.2 (0.29)*
Conscientiousness	61.8 (0.43)	62.0 (0.18)	61.9 (0.36)	61.8 (0.32)
Anxiety (N1)	10.4 (0.12)*	10.0 (0.05)	9.7 (0.10)*	9.8 (0.10)*
Angry hostility (N2)	7.4 (0.13)	7.4 (0.05)	7.5 (0.07)	7.4 (0.05)
Depression (N3)	7.9 (0.14)	7.8 (0.06)	7.9 (0.12)	8.1 (0.11)
Self-consciousness (N4)	9.5 (0.12)*	9.4 (0.05)	8.8 (0.10)*	8.6 (0.10)*
Impulsiveness (N5)	8.5 (0.12)	8.5 (0.06)	8.3 (0.10)	8.3 (0.09)
Vulnerability (N6)	9.3 (0.12)	8.9 (0.05)	8.5 (0.10)*	8.6 (0.09)*
Warmth (E1)	10.4 (0.11)	10.7 (0.05)	10.7 (0.10)	10.6 (0.09)
Gregariousness (E2)	9.5 (0.12)	9.7 (0.05)	9.8 (0.11)	9.7 (0.10)
Assertiveness (E3)	9.8 (0.12)	9.9 (0.06)	9.8 (0.12)*	9.6 (0.11)
Activity (E4)	9.1 (0.12)	9.4 (0.05)	9.6 (0.11)	9.2 (0.10)
Excitement-seeking (E5)	10.3 (0.12)	10.4 (0.05)	10.5 (0.10)	10.5 (0.10)
Positive emotions (E6)	10.0 (0.11)	9.9 (0.05)	10.1 (0.10)	9.9 (0.09)
Fantasy (O1)	9.9 (0.15)	10.0 (0.06)	9.8 (0.13)	9.7 (0.10)
Aesthetics (O2)	10.7 (0.14)	10.7 (0.06)	10.4 (0.12)	10.2 (0.11)
Feelings (O3)	10.7 (0.10)*	10.7 (0.04)	10.5 (0.09)*	10.3 (0.08)*
Actions (O4)	9.4 (0.12)	9.6 (0.05)	9.4 (0.10)	9.1 (0.10)
Ideas (O5)	8.8 (0.12)*	8.8 (0.06)	8.7 (0.12)*	8.6 (0.10)*
Values (O6)	10.7 (0.09)	10.8 (0.04)	10.7 (0.08)	10.4 (0.08)
Trust (A1)	9.7 (0.12)*	10.0 (0.05)	10.2 (0.09)*	10.3 (0.08)*
Straightforwardness (A2)	10.6 (0.14)*	10.9 (0.06)	10.9 (0.12)*	11.1 (0.10)
Altruism (A3)	11.1 (0.10)	11.3 (0.04)	11.3 (0.08)	11.3 (0.08)
Compliance (A4)	9.6 (0.11)	9.6 (0.05)	9.8 (0.09)*	9.8 (0.08)*
Modesty (A5)	10.3 (0.09)	10.4 (0.04)	10.5 (0.09)	10.9 (0.07)
Tender-mindedness (A6)	10.1 (0.11)	10.3 (0.05)	10.5 (0.09)*	10.8 (0.08)*
Competence (C1)	9.7 (0.10)	9.7 (0.05)	9.5 (0.10)	9.2 (0.08)
Order (C2)	10.3 (0.12)	10.2 (0.05)	10.0 (0.11)	10.1 (0.09)
Dutifulness (C3)	11.3 (0.09)	11.5 (0.04)	11.7 (0.08)	11.8 (0.08)
Achievement striving (C4)	10.0 (0.12)*	10.0 (0.05)	10.0 (0.10)	9.9 (0.09)
Self-discipline (C5)	10.3 (0.10)	10.5 (0.04)	10.7 (0.10)	10.7 (0.09)
Deliberation (C6)	10.2 (0.12)	10.2 (0.05)	10.0 (0.09)	10.1 (0.09)

Data are presented as adjusted mean (standard errors).

All means are controlled for age, age squared and education.

Wilk’s Lambda = 0.866, *P*<0.001, partial η2 = 0.047.

*P<0.05 compared to normal BMI groups (18.5–22.9kg/m^2^).

Multiple linear regression analysis was used to analyze the personality traits associated with BMI and waist circumference. In men, only the extraversion trait was positively associated with BMI (β = 0.032, *P*<0.05). Low dutifulness was associated with higher BMI (β = −0.135, *P*<0.05) and greater waist circumference (β = −0.381, *P*<0.05). In women, neuroticism was inversely associated with BMI (β = −0.026, *P*<0.05). Low openness to experience and high agreeableness were associated with an elevated BMI and a greater waist circumference (for BMI: β = −0.072, *P*<0.05 (openness to experience) and β = 0.068, *P*<0.05 (agreeableness); for waist circumference: β = −0.202, *P*<0.05 (openness to experience) and β = 0.227, *P*<0.05 (agreeableness)). Hostile and depressed individuals with lower self-consciousness and vulnerability were more likely to be overweight or obese, and experience greater central obesity. BMI and waist were also inversely associated with aesthetics, feelings, values, competence, and were positively associated with trust, modesty, and tender-mindedness (Table 4).

**Table 4:** Regression analyses of personality traits, body mass index (BMI) and waist circumference, men and women.

Variables	Men		Women	
	BMI	Waist	BMI	Waist
Age	0.082	0.250	0.111*	0.451*
Age squared	−0.001	−0.003	−0.002*	−0.002*
Education level	0.131	−0.512	−1.088*	−3.501*
Neuroticism	0.009	−0.039	−0.026*	−0.037
Extraversion	0.032*	0.047	0.009	0.034
Openness to experience	0.001	−0.068	−0.072*	−0.202*
Agreeableness	0.014	0.119	0.068*	0.227*
Conscientiousness	−0.003	−0.057	0.002	−0.017
Anxiety (N1)	0.035	0.062	−0.094*	−0.100
Angry hostility (N2)	0.054	0.199	0.150*	0.512*
Depression (N3)	−0.017	−0.075	0.156*	0.469*
Self-consciousness (N4)	−0.027	−0.300*	−0.257*	−0.827*
Impulsiveness (N5)	0.012	0.022	0.028	0.167
Vulnerability (N6)	−0.019	0.077	−0.188*	−0.446*
Warmth (E1)	0.083	0.198	−0.005	−0.113
Gregariousness (E2)	0.045	0.087	−0.032	−0.129
Assertiveness (E3)	0.041	0.135	0.033	−0.020
Activity (E4)	−0.068	−0.420*	−0.002	−0.071
Excitement seeking (E5)	0.053	0.066	0.081*	0.261*
Positive emotions (E6)	−0.037	0.008	0.008	0.185
Fantasy (O1)	−0.017	−0.102	−0.027	−0.043
Aesthetics (O2)	<0.001	−0.010	−0.073*	−0.362*
Feelings (O3)	0.081	0.037	−0.110*	−0.473*
Actions (O4)	−0.044	0.205	−0.073*	0.025
Ideas (O5)	−0.035	−0.090	0.037	0.137
Values (O6)	−0.019	−0.022	−0.227*	−0.458*
Trust (A1)	0.018	0.065	0.155*	0.656*
Straightforwardness (A2)	0.008	0.188	−0.002	0.037
Altruism (A3)	0.057	0.009	−0.031	−0.019
Compliance (A4)	−0.009	-0.039	0.022	0.031
Modesty (A5)	0.007	0.316	0.106*	0.253*
Tender-mindedness (A6)	0.064	0.150	0.176*	0.569*
Competence (C1)	0.104*	0.228	−0.168*	−0.296*
Order (C2)	−0.049	−0.265	−0.026	−0.092
Dutifulness (C3)	−0.135*	−0.381*	0.175*	0.220
Achievement striving (C4)	0.053	0.176	−0.060	−0.306
Self-discipline (C5)	−0.007	0.073	0.033	0.156
Deliberation (C6)	0.031	−0.071	−0.027	−0.046

*P<0.05 after controlling for age, age squared and education.

Low education levels were associated with increased BMI (β = −1.088, *P*<0.001) and increased waist circumference in women (β = −3.501, *P*<0.001). This association was not present in the men (β = 0.131, *P = *0.310 (BMI); −0.512, *P = *0.198 (waist circumference)). Income was inversely associated with BMI in women (β = −0.268, *P = *0.001, data not shown).

The results of the logistic regression analysis revealed that lower neuroticism was an important predictor of overweight and that low openness to experience was a significant predictor of obesity in women (OR 0.912, 95% confidence interval (CI) 0.834–0.996, *P* = 0.041 (neuroticism); OR 0.910, 95% CI 0.836–0.991, *P* = 0.030 (openness to experience)) (Table S1). In men, only the low self-consciousness variable was associated with overweight (OR 0.767, 95% CI 0.598–0.984, *P* = 0.037, details not shown).

## Discussion

We examined the association between personality traits and abnormal BMI in Korean men and women. Overweight and obese men scored higher on openness to experience and lower on conscientiousness compared with normal weight groups. Overweight and obese women scored lower on neuroticism and openness to experience and higher on agreeableness. Neuroticism was inversely associated with BMI in women. Lower openness to experience and higher agreeableness were associated with obesity and a greater waist circumference.

Neuroticism is the tendency to respond with negative emotions, and associated with poor physical health [25]. Highly neurotic individuals experience sympathetic hyperactivation and decreased immune function, have abnormal BMIs, and participate in unhealthy lifestyles [26]–[28]. The results of our study indicated that higher neuroticism was more likely to occur in underweight women. This finding has been identified in individuals with eating disorders and a desire for thinness [29], [30]. Women that are dissatisfied with their body image are more anxious and more likely to have eating disorders [31], [32].

Similar to findings for a Japanese population, overweight and obese women in our study scored lower in neuroticism [15]. However, the majority of the studies of personality traits and BMI have found that higher neuroticism is associated with overweight and obesity [10], [17], [19], [33]. Although neuroticism scores may be negatively associated with overweight and obesity in women, variables such as angry hostility and depression are likely to be positively associated with overweight and obese, and a larger waist circumference. Hostile individuals are less likely to exhibit dietary restraint and have an increased perception of hunger, which leads to weight gain, and depression is linked with metabolic syndrome [7], [34]–[36]. In our study population, anxiety, self-consciousness, and vulnerability were negatively associated with overweight and obesity. The effects of these variables most likely contributed to the finding that neuroticism was negatively associated with BMI. There were published studies that have found low neuroticism in overweight and obese groups, but different questionnaire was used and facets of neuroticism were not included.

Changes in openness to experience scores were also associated with abnormal BMI in both men and women. Underweight, overweight, and obese men were more likely to score higher in openness to experience, but women were more likely to score lower in this trait. The results for women were consistent with previous studies that found that lower openness to experience is likely to be associated with obesity and the presence of metabolic syndrome [6], [10], [37]. Openness to experience is a personality trait that helps an individual to overcome stress, so it may have a protective effect against inflammation and subsequent chronic diseases [38], [39]. Lower openness to experience may stimulate the production of stress hormones (e.g., cortisol), which may increase food craving and lead to weight gain [40]. Published studies did not find high openness associated with overweight or obesity, but low openness to experience was related to earlier depression onset in the elderly [41]. The presence or absence of a correlation between openness to experience and BMI remains to be determined.

In our study, agreeableness was positively associated with overweight and obesity in women. Being more agreeable is associated with overeating and elevated stress reactivity, which leads to greater consumption of snacks [17], [42]. Other studies have reported findings that conflict with our results; the lower agreeableness demonstrated by overweight and obese individuals is likely due to higher antagonism and hunger susceptibility [6], [7].

Overweight and obese men scored higher in extraversion. Increased warmth, gregariousness, and assertiveness contributed to this result. An unhealthy life style practiced by extraverted individuals that includes excessive and hazardous drinking may contribute to this association [43]. However, the subjects that participated in our study were not asked about social drinking, so further studies are necessary to evaluate this relationship.

Conscientiousness scores were inversely related to overweight and obesity in men. Lower order and dutifulness scores contributed to this result. Conscientiousness is associated with longevity and low mortality risk [44]. Individuals with low conscientiousness tend to be less concerned about their health and exhibit poor self-control and unhealthy behaviors that lead to the development of obesity, diabetes, and metabolic syndrome [9], [13], [18], [45], [46].

The results for the contribution of socioeconomic status revealed that women with lower educational levels were more likely to be overweight and obese, and to have a larger waist circumference. Income level was also inversely associated with BMI. Increased educational level was protective against the development of overweight and obesity because these women recognize the importance of choosing beneficial health behaviors (e.g., better dietary choices). A lower income level is related to greater animal fat intake and decreased healthy food consumption [47], [48].

There are some limitations in our study. The total number of men was relatively small compared with the total number of women, so it was difficult to generalize the findings for both genders. Adjusted mean values that were significant in a univariate analysis were not significant when they were included in the linear or logistic regression analyses, probably because of the small sample sizes. The use of the short version of the NEO-PI-R may have also imposed some limitations on the interpretation of the contribution of individual facets of personality traits. Different results may have been obtained if we had used the original version of the questionnaire, but the size (240 items) imposed limits on the use of this survey instrument in a population with elderly participants. There were also many factors that contribute to body weight that we did not consider in this study (e.g., eating habits, other socioeconomic status variables) [11], [49]. Change in body weight also was not considered, so the relationship between weight regain and behavioral factors was not included in the analysis [50]. Baseline measurements of personality traits were not available, so the presence of reverse causality may have affected the findings.

To our knowledge, this study is the first to use the FFM to analyze the relationship between personality traits and underweight, overweight, and obesity in a Korean population. The results of this study were somewhat different from the results of similar studies performed in populations from Western countries. However, previous studies have also obtained contrasting results when comparing the same personality traits, so it is difficult to determine which traits are more strongly associated with underweight, overweight, or obesity. Ethnic or national differences may contribute to the differences seen in the results. Longitudinal and worldwide studies will help to determine which traits are more or less dependent on specific study population characteristics.

In conclusion, personality traits were linked with abnormal BMI in this Korean population. Specific traits may be more strongly associated with underweight, while others are more specific for overweight and obesity. Understanding the contribution of the psychological aspects of these traits will help us to improve the approach used for the prevention and treatment of underweight, overweight, and obesity.

## Supporting Information

Table S1 **Personality traits predicting underweight, overweight, and obesity in women.**
(DOC)
