# Supplementary material for: Personality Traits and Body Mass Index in a Korean Population
Source: PLoS One. 2014 Mar 5;9(3):e90516. doi: 10.1371/journal.pone.0090516 (PMC3944006; doi:10.1371/journal.pone.0090516)
Supplement: Table S1 — Personality traits predicting underweight, overweight, and obesity in women. (DOC) [file pone.0090516.s001.doc]

Supplementary Table 1. Personality traits predicting underweight, overweight, and obesity in women.

|  | Underweight (n=273)  vs. normal (n=1452) | | Overweight (n=363)  vs. normal (n=1452) | | Obese (n=459)  vs. normal (n=1452) | |
| --- | --- | --- | --- | --- | --- | --- |
| Variables | OR | 95% CI | OR | 95% CI | OR | 95% CI |
| Age | 1.057 | 0.938–1.190 | 1.164 | 1.076–1.260^*^ | 1.312 | 1.218–1.414^*^ |
| Age squared | 1.000 | 0.999–1.002 | 0.999 | 0.998–1.000^*^ | 0.998 | 0.997–0.999^*^ |
| Education | 0.993 | 0.984–1.003 | 0.985 | 0.965–1.006 | 1.005 | 0.995–1.016 |
| Neuroticism | 0.915 | 0.827–1.013 | 0.912 | 0.834–0.996^*^ | 0.949 | 0.868–1.038 |
| Extraversion | 0.862 | 0.785–0.946^*^ | 1.074 | 0.988–1.168 | 1.064 | 0.981–1.154 |
| Openness to experience | 1.074 | 0.973–1.185 | 1.026 | 0.941–1.119 | 0.910 | 0.836–0.991^*^ |
| Agreeableness | 0.986 | 0.908–1.071 | 0.963 | 0.890–1.043 | 1.001 | 0.923–1.085 |
| Conscientiousness | 0.990 | 0.909–1.078 | 1.016 | 0.940–1.097 | 1.076 | 0.997–1.162 |
| Anxiety (N1) | 0.983 | 0.851–1.135 | 1.125 | 0.994–1.274 | 1.095 | 0.965–1.243 |
| Angry hostility (N2) | 1.088 | 0.942–1.255 | 1.200 | 1.055–1.364^*^ | 1.042 | 0.919–1.181 |
| Depression (N3) | 1.148 | 0.995–1.325 | 1.139 | 1.004–1.292^*^ | 1.162 | 1.025–1.317^*^ |
| Self-consciousness (N4) | 1.195 | 1.036–1.378^*^ | 1.063 | 0.936–1.207 | 1.011 | 0.891–1.147 |
| Impulsiveness (N5) | 1.114 | 0.967–1.282 | 1.096 | 0.966–1.243 | 1.116 | 0.983–1.268 |
| Warmth (E1) | 1.286 | 1.109–1.490^*^ | 0.944 | 0.829–1.075 | 0.898 | 0.791–1.020 |
| Gregariousness (E2) | 1.178 | 1.034–1.342^*^ | 0.927 | 0.827–1.038 | 0.915 | 0.819–1.023 |
| Assertiveness (E3) | 1.145 | 1.010–1.296^*^ | 0.967 | 0.864–1.082 | 1.090 | 0.975–1.218 |
| Activity (E4) | 1.290 | 1.127–1.476^*^ | 0.986 | 0.870–1.117 | 0.955 | 0.847–1.077 |
| Excitement-seeking (E5) | 1.147 | 1.004–1.310^*^ | 0.890 | 0.791–1.001 | 0.923 | 0.821–1.037 |
| Fantasy (O1) | 0.986 | 0.873–1.113 | 0.957 | 0.857–1.068 | 1.144 | 1.023–1.279^*^ |
| Aesthetics (O2) | 0.930 | 0.829–1.043 | 0.966 | 0.869–1.074 | 1.059 | 0.953–1.178 |
| Feelings (O3) | 0.962 | 0.833–1.112 | 1.008 | 0.887–1.144 | 1.108 | 0.980–1.253 |
| Actions (O4) | 0.974 | 0.859–1.104 | 0.964 | 0.862–1.078 | 1.058 | 0.949–1.180 |
| Ideas (O5) | 0.911 | 0.800–1.037 | 1.005 | 0.897–1.125 | 1.126 | 1.004–1.264^*^ |
| Trust (A1) | 1.050 | 0.925–1.193 | 1.094 | 0.973–1.229 | 1.050 | 0.934–1.180 |
| Straightforwardness (A2) | 1.056 | 0.942–1.183 | 0.973 | 0.875–1.082 | 0.916 | 0.824–1.017 |
| Altruism (A3) | 1.051 | 0.902–1.223 | 1.032 | 0.897–1.187 | 0.956 | 0.832–1.098 |
| Compliance (A4) | 0.937 | 0.827–1.061 | 1.077 | 0.956–1.214 | 1.022 | 0.906–1.152 |
| Modesty (A5) | 0.529 | 0.912–1.198 | 0.986 | 0.868–1.121 | 1.146 | 1.007–1.304^*^ |
| Competence (C1) | 0.985 | 0.860–1.129 | 0.964 | 0.856–1.085 | 0.898 | 0.797–1.011 |
| Order (C2) | 0.976 | 0.855–1.116 | 0.914 | 0.813–1.028 | 0.871 | 0.775–0.979^*^ |
| Dutifulness (C3) | 0.966 | 0.831–1.124 | 1.050 | 0.918–1.200 | 0.945 | 0.829–1.078 |
| Achievement striving (C4) | 0.962 | 0.837–1.105 | 0.909 | 0.799–1.034 | 0.853 | 0.753–0.966^*^ |
| Self-discipline (C5) | 1.078 | 0.943–1.232 | 0.969 | 0.860–1.092 | 0.896 | 0.794–1.012 |

OR, odds ratio; CI, confidence interval

^*^ *P* < 0.05
